# Effect of dental restorative materials on total antioxidant capacity and calcium concentration of unstimulated saliva

**DOI:** 10.4317/jced.53272

**Published:** 2017-01-01

**Authors:** Gholam H. Ramezani, Mona-Momeni Moghadam, Mohammad-Ali Saghiri, Franklin Garcia-Godoy, Armen Asatourian, Mohsen Aminsobhani, Mark Scarbecz, Nader Sheibani

**Affiliations:** 1 BDS, MS, DDS, Associate professor, Department of Pediatric Dentistry, Dental Branch, Islamic Azad University, Tehran, Iran; 2 DDS Visiting researcher, Angiogenesis and Regenerative Group, Dr. H. Afsar Lajevardi Research Cluster, Shiraz, Iran; 3 BSc, MSc, PhD, Wisconsin institute for Medical Research and Department of Ophthalmology & Visual Sciences, University of Wisconsin School of Medicine and Public Health, Madison, WI, USA; 4 DDS, MSc, PhD, Bioscience Research Center, Health Science Center, College of Dentistry, University of Tennessee, Memphis, TN, USA; 5 DDS, Clinical instructor, Angiogenesis and Regenerative Sector, Dr. H. Afsar Lajevardi Research Cluster, Shiraz, Iran; 6 DDS, MSc, Associate professor, Endodontic Department, School of Dentistry, Tehran University of Medical Sciences and AJA University of Medical Sciences, Tehran, Iran; 7 DDS, PhD, Professor, Bioscience Research Center, Health Science Center, College of Dentistry, University of Tennessee, Memphis, TN, USA; 8 BSc, MSc, PhD, Professor, Departments of Ophthalmology & Visual Sciences, University of Wisconsin School of Medicine and Public Health, Madison, WI, USA

## Abstract

**Background:**

To evaluate the effect of dental amalgam and composite restorations on total antioxidant capacity (TAC) and calcium (Ca) ion concentration of unstimulated saliva.

**Material and Methods:**

Forty-eight children aged 6-10 years selected and divided into three groups of sixteen (8 males, 8 females). In group A and B, samples consisted of two class II dental composite or amalgam restorations, while in group C samples were caries-free (control group). Unstimulated saliva from all samples was collected and TAC was measured by spectrophotometry using an adaptation of 2, 2’-azino-di-(3-ethylbenzthiazoline-6-sulphonate) (ABTS) assay. The Ca ion level was estimated by an auto- analyzer. Data were analyzed with one- and two-way ANOVA test, at a *p*<.05 level of significance.

**Results:**

Composite samples showed significantly higher TAC and lower Ca ion levels compared to amalgam and caries-free samples (*p*<.05). The TAC values showed only significant difference between groups (*p*<.05), while the Ca ion results showed significant differences within and between groups (*p*<.05).

**Conclusions:**

Dental composite restorations increased TAC and decreased Ca ion levels more than amalgam restorations in saliva. Gender is an effective factor in changes induced in oral cavity as females showed more emphatic reaction to dental filling materials than males.

**Statement of Clinical Relevance:**

Patients who have dental restorations, especially dental composites, should pay more attention to their dental hygiene, because dental restorations can increase oxidative stress and decrease Ca ion level in saliva, which might jeopardize remineralization process of tooth structures after demineralization.

** Key words:**Amalgam, caries, composite, saliva, total antioxidant capacity.

## Introduction

Saliva is one of the most important human body fluids, which is mainly secreted in the oral cavity from three pairs of major salivary glands including parotid, submandibular and sublingual and many minor salivary glands (1). Saliva has multiple functions inside the oral cavity including lubrication, digestion, taste, pH buffering, immunological activity, mechanical cleansing of carbohydrates, post-eruptive maturation of enamel, remineralization of tooth structures after demineralization and vocalization (2,3). The secretion of saliva in a healthy adult is about 500-1500 ml/per day, which is produced at a rate of approximately 0.5 ml/min (4). The composition of saliva includes inorganic molecule such as ions and organic substances such as proteins, non-proteins, and hormones (5,6). The inorganic ions in saliva are mainly sodium (Na), potassium (K), calcium (Ca), phosphate (P), magnesium (Mg), and bicarbonate (HCO3), which contributes to buffer activity, post-eruptive maturation of enamel, and remineralization of tooth structures after demineralization. Among these ions, Ca has a great effect in remineralization of demineralized tooth structures and is detectable in greater amount in unstimulated saliva than stimulated saliva. In unstimulated saliva, the main sources of Ca secretion are submandibular and sublingual salivary glands, while in stimulated saliva parotid glands produce a greater amount (7). The presence of Ca in saliva is of great importance as saliva contains proteins such as statherin and proline rich proteins (PRPs) that bind to Ca to inhibit the precipitation of Ca and promote the remineralization of tooth structures (8).The proteins and polypeptides of saliva includes digestive enzymes including α-amylase, plasma proteins such as albumin and transferrin, immunological proteins like secretory-IgA, plasma derived IgG and IgM, lysozyme, lactoferrin, cyctatin groups, PRPs (antimicrobial activity), and hystatin (antifungal activity), statherin, and mucins groups act as lubricant (8). Saliva contains organic non-protein molecules such as uric acid with antioxidant activity (9), creatinine, bilirubin (10), glucose, lactose, amino acids, and lipids such as cholesterol and mono/di glycerides (5,6,11,12). The other organic components of saliva are steroids, non-steroids, and protein hormones including cortisol, testosterone, estradiol, progesterone, and aldosterone (8). Due to its wide range of components, safe sampling, non-invasive collection, and easy storage of saliva is a valuable diagnostic tool for many diseases and conditions such as oral diseases, benign and malignant oral tumors, Sjögren syndrome, Beçhet syndrome, bacterial and viral infections, drug abuse, DNA tests, and cancers such as breast cancer (13-19). One of the diagnostic biomarkers of saliva is the total antioxidant capacity (TAC), which includes both enzymatic and non-enzymatic antioxidants that are the first line of defense against oxidative stress and several oral and systemic diseases (20). The enzymatic antioxidants include catalase, superoxide dismutase, and glutathione peroxidase. The non-enzymatic antioxidants include ascorbic acid (vitamin C), β-carotene, α-tocopherol (vitamin E), and flavonoids (20,21). TAC is a biomarker comprising all saliva antioxidants and has a great clinical significance as many oral and systemic pathological conditions undergo remarkable changes. Therefore, TAC can be used for evaluation of several diseases and syndromes such as dental caries (22), periodontal disease (23), diabetes mellitus (24), and Down syndrome (25).

According to these facts, Ca is vitally important ion in saliva and TAC is a valuable diagnostic biomarker for evaluation of several conditions. However, the changes in these components have not been measured in relation with dental restorations. Therefore, the present study evaluated the effects of dental amalgam and composite restorations on TAC and Ca ion concentration. It is hypothesized that dental amalgam and composite restorations might change the oral cavity environmental condition in a way that these components of saliva can be influenced.

## Material and Methods

-Sample preparation 

This study was performed in accordance with guidelines of 64th WMA General Assembly, Fortaleza, Brazil, October 2013, in a private dental office in Tehran, Iran. Forty-eight healthy male and female children between 6-10 years of age were selected for this study and divided into three groups of sixteen including 8 males and 8 females. In group A, children had two dental composite restorations; in group B, children had two dental amalgam restorations; and in group C, children were caries-free and did not have any restorations; this group served as the control group. The inclusion and exclusion criteria were similar to those previously reported by others (22,26) as follows:

▪General inclusion criteria: 1) all children were 6-10 years old and their parents agreed with the terms and conditions of this study by signing consent forms prepared in accordance with the Helsinki guidelines. 2) Children should not have any local or general medical diseases or conditions, especially those which affect saliva secretion. 3) Children should be permanent resident of the same city where the study was performed to control the water consumption (municipal water). 4) Children underwent diet control for not consuming any sea food for at least one week before saliva collection. 5) Children should not have any missing and supernumerary teeth.

▪Specific inclusion criteria: 1) the dental restorations in the experimental groups should be two dental composites (group A) or amalgam (group B) class II restorations; children should be aged between 12-18 months (1-1.5 years after restoration placement). 2) The dental restorations should be in mandibular D and/or E primary molars with no liners or base materials applied beneath the filling materials. 3) Children in group A and B should not have any active or arrested dental caries. 4) The caries-free children in the control group should not had any decay, missing, and filling surfaces (DMFs = 0).

▪Exclusion criteria: 1) Children who had local or systemic diseases for conditions affecting the oral cavity environment and saliva production. 2) Children who had missing or supernumerary teeth. 3) Children who had more than two dental restorations, or two not identical restorations (one composite and one amalgam), or restorations on teeth other than primary molars. 4) Children with two identical dental restorations and active, arrested, or secondary dental carries.

The unstimulated saliva was collected from all children under aseptic conditions. According to a previous study (22), saliva was allowed to be accumulated in the children’s mouth for 2-3 minutes and then 2 ml of saliva was aspirated with a sterile syringe from the floor of the mouth. The collected samples were stored in 4 ºC temperature and transferred to the laboratory and underwent TAC and Ca ions concentration measurement.

-Measurement of total antioxidant capacity:

The saliva TAC measurement was performed using spectrophotometry by adaptation of 2, 2’-azino-di-(3-ethylbenzthiazoline-6-sulphonate) (ABTS) assay (27), using Biochrom Asys Expert kit. The pyridoxal is used as a substrate and undergone oxidation, which produces pyridoxic acid and hydrogen peroxide. By the action of peroxidase, hydrogen peroxide is reduced to water and the chromogen ABTS is oxidized to a blue-greenish-dyed product. The collected saliva from samples includes antioxidants that can inhibit the production of ABTS. Sample saliva (0.5 ml) was added to the plates and the amount of ABTS produced was measured by reading the absorbance at 405 nm in a plate reader (Biochrom Asys Expert 96 Microplate Reader, Biochrom, Holliston, MA, USA).

-Measurement of Ca ion concentration.

The Ca ion concentration was estimated by an autoanalyzer (Auto analyzer (BT 3000 Plus, Biote- canica Instruments SpA, Italy).

-Statistical analysis

Data were analyzed with one- and two-way ANOVA tests. Correlations between TAC and Ca ion were analyzed with Pearson’s correlation coefficient. Data was analyzed using SPSS V22.0 (IBM, Armonk, NY USA). Statistical significance was set at *p*<.05.

## Results

**Figure 1:**
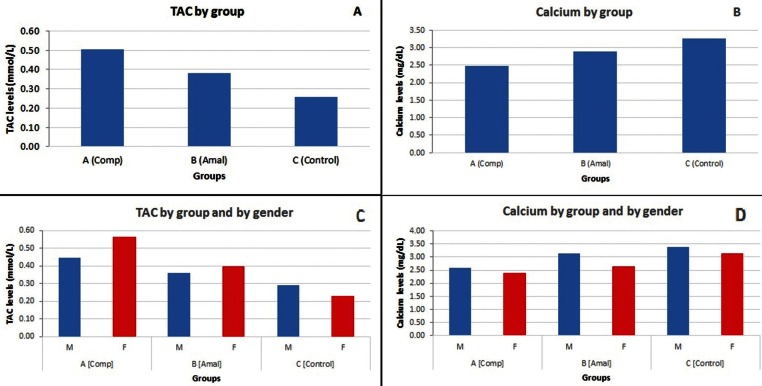
A) The comparison of TAC by Groups; B) The comparison of Ca ion by group; C) The comparison of TAC by group and gender; D) The comparison Ca by group and gender.

-Total antioxidant capacity:

The means and standard deviations of TAC levels in samples’ saliva were: 0.45 ± 0.06 (composite, males), 0.57 ± 0.07 (composites, females), 0.36 ± 0.05 (amalgam, males), 0.40 ± 0.05 (amalgam, females), 0.29 ± 0.04 (caries-free, males), and 0.23 ± 0.06 (caries-free, females), respectively. The highest amount of TAC was seen in composite female samples, while the lowest was in caries-free females. In a 2-way ANOVA (TAC by group and by gender), the main gender effect was not significant (*p*>.05), indicating no difference in male and female. The main effect of group was significant (*p*<.05), indicating differences across groups. The interaction effect of group x gender was also statistically significant (*p*<.05), indicating that the gender effect varies by group with females differing significantly from males for the group A (Composite) and C (Caries free) groups (*p*<.05) (Fig. 1A,C).

-Ca ion concentration:

The means and standard deviations of Ca ion levels in sample saliva were: 2.59 ± 0.45 (composite, males), 2.38 ± 0.37 (composites, females), 3.12 ± 0.21 (amalgam, males), 2.65 ± 0.29 (amalgam, females), 3.38 ± 0.40 (caries-free, males), and 3.14 ± 0.41 (caries-free, females), respectively. The highest amount of Ca ion was noticed in caries-free males and the lowest value was seen in females with composite restorations. In a 2-way ANOVA (Ca ion by group and by gender), the main gender and group effects were significant (*p*<.05), indicating differences by group and by gender. The interaction effect of group x gender was not statistically significant (p>.05), indicating that the sex effect does not vary by Group (Fig. 1 B,D).

**Figure 2:**
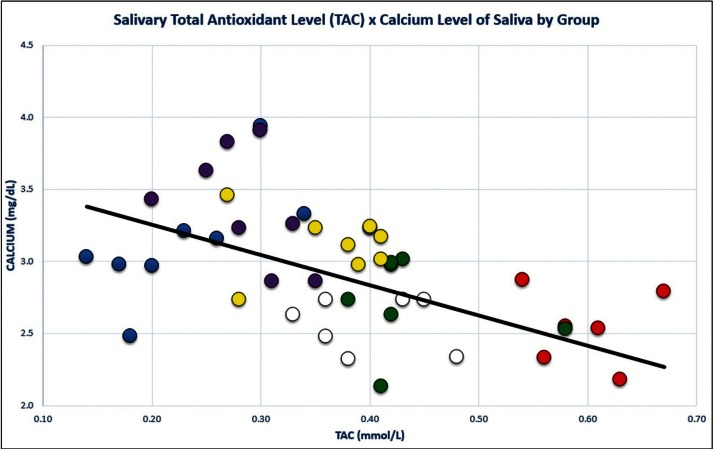
The correlation of TAC with Calcium. (r= -.524, *p*<.05) by Group. b = -2.10 (Slope of Regression Line; r2=.27). Key: Green: Males, Group A (Amalgam); Yellow: Males, Group B (Composite); Purple: Males, Group C (Caries free); Red: Females, Group A (Amalgam); White: Females, Group B (Composite); Blue: Females, Group C (Carries free).

Overall, there was a correlation between TAC and Ca ion (r=-.524, *p*<.05). However, within experimental groups the correlations (gender x group) were not statistically significant (*p*>.05) (Fig. 2).

## Discussion

TAC is one of the valuable saliva biomarkers, which is used for detecting and monitoring many local and general diseases (22-25). Ca ion also is of utmost importance in saliva as it has great influence on remineralization of demineralized tooth structures (7,8). Dental composites are dental materials widely used for restorative purposes due to their esthetic and tooth adhering abilities (28). However, the application of dental amalgam has been reduced due to growing concerns regarding the toxicity of these traditionally used materials (26). Therefore, the present study evaluated the effect of dental composite or amalgam restorations on TAC and Ca ion levels in saliva.

These results indicated that saliva of children having dental composite restorations had significantly more TAC levels than dental amalgam and caries-free samples (Fig. 1A). This increase in levels of TAC can be attributed to dental composite degradation products. In aqueous environment, such as the oral cavity, water molecules can diffuse into the composite materials and cause chemical degradation of dental composites (29,30). About 30 different substances can be released from polymerized composites including major monomers, comonomers, additives, and reaction products, which have cytotoxic effects on living tissues (31-33). The major monomers include bisphenol A glycidyl methacrylate (BisGMA), triethylene glycol dimethacrylate (TEGDMA), urethane dimethacrylate (UDMA), and bisphenol A (BPA). These monomers can be released even one year after composite light curing time (34). This is consistent with the results of present study. The dental composites were performed 1-1.5 year prior to the saliva sampling.

There are three general effects caused by unbound monomers. First, they may affect caries forming bacterial growth resulting in secondary caries. Second they are cytotoxic causing dental pulp reaction and gingival tissue retraction. Third they can induce allergic reactions in 0.7-2 % of the population (35). Among these monomers, TEGDMA was shown to be the major monomer released from resin adhesives and composites, which is capable of influencing cellular metabolic state, lipid turnover, causing deletion of large sequences of a DNA molecule, and inducing apoptosis in gingival fibroblasts (36,37). These effects of TEGDMA is mainly attributed to its effect on production of reactive oxygen species (ROS) due to mitochondrial damage, which is claimed to be the major reason for TEGDMA-induced cell death (38). The production of ROS resulting from unbound and non-polymerized monomers such as TEGDMA can explain the elevation of TAC levels in saliva samples. This observation is consistent with previously demonstrated results indicating that TAC, albumin, and uric acid are important biomarkers for monitoring the oxidative stress (OS) in the oral cavity (39). ROS is also produced during periodontal tissue destruction caused by microorganism activity, which lead to elevation of antioxidant defense system response in the oral cavity (40).

The elevation of TAC was also observed in female and male children with dental amalgam restorations (Fig. 1C). Dental amalgam is a mixture of mercury with other alloys including silver, copper, tin, zinc, indium, palladium, lead, cadmium, and antimony (26). Many of these metallic ions, especially mercury, silver, and tin are released from dental amalgam in dynamic environment of oral cavity for a long time after teeth restorations (26,41). Among these components, mercury produces ROS, which initiates the oxidative stress and cellular damages (42,43). Moreover, silver nanoparticles (AgNPs) have been used as anti-cancer agents due to generation of ROS, which causes DNA damage (44). The generation of ROS caused by the release of metallic ions from dental amalgam can explain the elevation of TAC levels in the present study. This observation is not consistent with the results of Pizzichini *et al.* (45) who claimed that mercury release from dental amalgams decreased the amount of antioxidant levels in saliva. These authors evaluated the level of total antioxidant activity (TAA) in patients with 0-10 amalgam restorations or surfaces. This difference can be explained by the methodology utilized by these authors, as the sample size for amalgam restorations were not equal and most of the patients with two amalgam restorations showed higher antioxidant values than patients with more restorations.

Ca ion levels in the experimental groups were decreased in children with composite and amalgam restorations, while composite restorations induced more reduction than amalgam (Fig. 1B). Ca level also changed according to alterations in the oral cavity. Some have indicated that Ca ion level was decreased in saliva of children with active caries compared to caries-free children (22). This outcome is consistent with the results of the present study as the presence of dental restorations reduced the amount of Ca ion in saliva compared to the control group. The reduced Ca and P ion levels were attributed to the participation of Ca and P in the remineralization process (22). However, in the present study it seems that the reason for Ca ion reduction is the oxidative stress caused by the generation of ROS due to release of substances from dental composite and amalgam restorations. It is indicated that oxidative stress besides imposing damage to the cell DNA, it can also interfere with cellular functions such as protein synthesis. Previous studies showed that oxidative stresses in the oral cavity can reduce the levels of cAMP and cGMP second messengers controlling the function of salivary gland cells (46). The lower amount of these messengers can lead to reduction of the flow rate and protein concentration in saliva (47). In saliva, Ca ion mostly binds to proteins such as statherin and PRPs, which inhibit the precipitation of Ca (8). According to this fact, any reduction in production of these proteins can increase the Ca precipitation, which might diminish the detectable amount of Ca in saliva. The decrease in secretion of saliva proteins due to ROS generation might explain the lower levels of Ca in saliva of patients with dental composite and amalgam restorations compared to caries-free patients. In addition, it seems that dental composites can induce ROS generation more than amalgam restorations, which can explain the significantly higher reduction of Ca in these patients. 

The other outcome of this study was the comparison of TAC and Ca ion levels within each group, which showed that females had higher TAC and lower Ca ion levels compared to males (Fig. 1C and D). These results showed that gender was an influential factor on TAC and Ca ion levels. Therefore, gender is an important factor in the secretion and flow rate of saliva. Some authors have reported that the diminished secretion of saliva in female is attributed to the smaller size salivary glands and hormonal pattern compared to males (48). The different hormonal pattern between female and male patients might explain the higher values of TAC and lower Ca ion levels in females.

## Conclusions

According to outcomes of this study, the following conclusions can be drawn:

• Dental composite and amalgam restorations increased the amount of TAC levels in saliva by trigging the oxidative stress in the oral cavity. Oxidative stresses occurred due to ROS generation caused by the releasing materials from dental restorations with time. The releasing components from dental composite restorations can trigger the oxidative stress more than the dental amalgam. This issue should be considered in future when manufacturing dental restorative materials to decrease or moderate the ROS generating capacity of these materials.

• The Ca ion levels in saliva can also diminish due to the increased oxidative stress produced by dental restorative materials. Dental composites reduced Ca ion levels of saliva more than dental amalgam. The lower level of Ca ion in saliva can jeopardize remineralization of tooth structures after demineralization. This effect of dental materials requires dental clinicians to encourage their patients to pay more attention to their dental hygiene after dental restorations.

• Gender influences the changes induced in oral cavity, as females showed more emphatic reaction to dental filling materials than males.
